# The Applications of Machine Learning to Prognostic Modeling for Human Immunodeficiency Virus: A Protocol for a Scoping Review

**DOI:** 10.1002/hsr2.71701

**Published:** 2025-12-29

**Authors:** Sadie Gilliland, Lawrence Mbuagbaw

**Affiliations:** ^1^ Faculty of Science McMaster University Hamilton Ontario Canada; ^2^ Department of Health Research Methods, Evidence, and Impact McMaster University Hamilton Ontario Canada; ^3^ Biostatistics Unit, Father Sean O'Sullivan Research Centre St Joseph's Healthcare Hamilton Hamilton Ontario Canada; ^4^ Department of Anaesthesia McMaster University Hamilton Ontario Canada; ^5^ Department of Pediatrics McMaster University Hamilton Ontario Canada; ^6^ Centre for Development of Best Practices in Health (CDBPH) Yaoundé Central Hospital Yaoundé Cameroon; ^7^ Department of Global Health, Division of Epidemiology and Biostatistics Stellenbosch University Cape Town South Africa

**Keywords:** artificial intelligence, disease progression, human immunodeficiency virus, machine learning, prognosis

## Abstract

**Background and Aims:**

As of late 2023, an estimated 39.9 million people are living with HIV, placing strain on healthcare systems. Machine learning (ML), a branch of artificial intelligence, enables systems to improve performance through data‐driven learning without explicit programming. HIV prognosis is influenced by clinical, epidemiological, and psychosocial factors, and ML algorithms have the potential to integrate these determinants efficiently. This can provide valuable insights into disease progression and risk assessment in terms of viral load, CD4 cell count, treatment initiation, treatment adherence, hospitalization, acquired immunodeficiency syndrome diagnosis, quality of life and mental health. This protocol outlines the existing applications of ML to prognostic modeling in the context of HIV, highlighting how ML can equip physicians with rapid and accurate predictions of disease progression, thereby informing treatment decisions such as clinical prescriptions and social support plans, and optimizing patient outcomes.

**Methods:**

The protocol follows the *Preferred Reporting Items for Systematic Reviews and Meta‐Analyses Extension for Scoping Reviews (PRISMA‐ScR)* framework. A search strategy has been developed for Medline (PubMed) and will be adapted for searches in Embase, Web of Science, Scopus, IEEE Xplore, and ACM Digital Library. The study selection and data extraction will be conducted in duplicate. The methods for the scoping review are prespecified to ensure transparency.

**Discussion:**

The proposed scoping review will identify effective model types, data inputs, and applications of ML in the context of HIV prognosis. While ML has been integrated into various aspects of HIV research, few studies have focused on predicting prognosis. This review aims to synthesize current uses of ML in prognostic modeling and highlight gaps within the existing technology. The findings from this review will support the development of future ML models that can inform clinical decision‐making, and, in turn, optimize patient care, improve resource allocation, and enhance public health responses to the ongoing HIV epidemic.

## 1 Background

At the end of 2023, an estimated 39.9 million people were living with HIV, with 65% residing in the World Health Organization (WHO) African Region [1]. HIV is transmitted through bodily fluids and secretions, which contributes to the vulnerability of virus contraction for high‐risk groups including sex workers, men who have sex with men, and people who inject drugs [2, 3]. While the proposed review examines HIV prognosis within the general population, these epidemiological insights offer important context for the creation of data‐driven prognostic evaluations.

Antiretroviral therapy (ART) is the primary treatment for HIV infection and, with proper use, is highly effective in suppressing the virus and managing the infection [4]. Access to ART is limited by insufficient supplies of antiretrovirals, particularly in low‐income countries, and barriers to accessing ART services persist even in regions where medications are readily available [4]. Without treatment, HIV will progress to AIDS and eventually lead to patient death [5]. Since the introduction of ART, HIV is no longer considered a fatal disease, and with prompt diagnosis and treatment, life expectancy is now relatively close to that of individuals without HIV [6]. When patients have access to treatment, proper adherence is required over 95% of the time for ART to maintain full viral suppression [7]. Poor adherence will result in poor prognosis, higher morbidity and mortality, and a higher risk of developing resistance to ART [7]. Therefore, considerations regarding ART access and adherence must be taken into account to accurately determine patient prognosis.

The HIV care cascade refers to the patient pathway, starting with HIV diagnosis and ultimately determining patient outcomes [8]. After receiving an HIV diagnosis, patient engagement with care facilitates timely initiation of treatment, optimal ART adherence, and sustained retention to the treatment plan. In combination, these factors result in optimal prognostic outcomes, highlighting the importance of considering each step of the HIV care cascade when predicting HIV prognosis [8].

Opportunistic infections are caused by bacteria, fungi, and viruses that typically cause mild effects in healthy individuals but can be life‐threatening in immunocompromised individuals [9]. Within populations of people living with HIV, these conditions are a major determinant of HIV prognosis given that they are the primary cause of severe symptoms [10]. Due to the shared transmission pathways, comorbidities commonly associated with HIV include tuberculosis, hepatitis B and hepatitis C [11]. Given these associations, predicting the risk of poor outcomes and the factors associated with poor outcomes can inform prevention strategies in the form of proactive healthcare measures to reduce the incidence of opportunistic infections and manage comorbidities thereby, supporting optimal care for people living with HIV. Such predictions could be informed by machine learning (ML) techniques.

ML is a branch of artificial intelligence that leverages systems to “learn” and improve performance without explicit programming [12]. By employing numerous algorithmic models and statistical methods, ML is able to solve complex problems by drawing information from data instead of relying on specialized code [13]. These models improve their performance and “intelligence” by training on large‐scale data sets, enabling them to recognize patterns more effectively [12]. In relation to healthcare, ML has been successfully implemented to identify trends and develop disease prediction models [13]. ML shows promise in enhancing public health initiatives by utilizing large data sets to offer insights into epidemiology factors and risk assessments [14]. Therefore, the potential applications of ML within HIV healthcare lie in its ability to identify high‐risk groups, determine the best strategy to prevent HIV transmission, develop optimal treatment plans, and predict the prognosis of individuals living with HIV.

As per the No Free Lunch Theorem, there is no single ML algorithm that is optimal for every analysis setting [15]. Rather, algorithm design and complexity must be specific to the data set to avoid overfitting and to facilitate the most accurate predictions [15]. For prognostic modeling in the context of COVID‐19, an infectious disease with some parallels to HIV, it has been shown that tree‐based classifiers provide a greater area under the receiver operating characteristic curve (AUROC) when compared to regression, margin, neural network, and instance‐based algorithms [16]. A greater AUROC value suggests that the ML model can better distinguish between classes with fewer errors. This aligns with other research indicating that supervised classification algorithms are a promising tool for accurately predicting risk and are therefore a valuable option for healthcare prognostics [17].

The literature indicates the most prominent area of ML implementation within HIV care to be within the field of prevention. Key research within this field stems from the identification of suitable candidates for preexposure prophylaxis [18]. A study by Marcus and colleagues published in 2019 created a least absolute shrinkage and selection operator (LASSO) regression which aimed to identify preexposure prophylaxis candidates from electronic health records and showed promising performance [19].

While less developed, several studies have explored the efficacy of utilizing ML algorithms to predict various aspects of HIV prognosis. Zhan and colleagues investigated outcomes for hospitalized patients with comorbid *cryptococcal* infection with the aim of reducing in‐hospital mortality rates [20]. This study trained ten ML models and found random forests and support vector machine models to be promising in their ability to predict patient prognosis. A study by Rivero‐Juárez and colleagues created a model to identify factors which should be accounted for in criteria used for prioritization of treatment for patients with HIV and hepatitis C coinfection [21]. This model performed well with an AUROC of 0.802. Prosperi and colleagues employed Cox regression and random survival forests to predict time to virologic failure for people living with HIV undergoing a new therapy regimen [22]. The results indicate that random survival forests are a promising tool to predict this metric. These algorithms have demonstrated success in their ability to accurately calculate prognosis and show promise for the implementation of a greater scale, universal model. By including clinical determinants, comorbidities and social factors as input data, an ML model could provide a more comprehensive and accurate prediction of patient prognosis. Based on the algorithm's output, physicians could have rapid access to the information necessary for developing a personalized treatment plan and optimizing patient health.

While there are reviews focusing on the use of ML within certain aspects of HIV care, notably prevention, the literature is lacking a synthesis of current ML applications within prognostic modeling for HIV. The effects of viral load, CD4 cell count, treatment initiation, treatment adherence, hospitalization, and mental health on HIV prognosis are critically important within HIV research to prevent disease progression and optimize quality of life for people living with HIV. This protocol outlines a review which aims to address this gap by identifying current applications of HIV prognostic modeling, key data inputs, and highlighting areas in need of further model development.

## 2 Objectives

The main objective of this scoping review is to summarize the evidence on practical applications of ML algorithms to prognostic modeling in the context of HIV. Specifically, the review will identify the types of ML models that have been used in HIV prognosis, the most informative data types for predicting HIV outcomes and opportunities of future implementation ML in HIV prognosis.

## 3 Methods

This protocol has been designed according to the Preferred Reporting Items for Systematic Reviews and Meta‐Analyses Extension for Scoping Reviews (PRISMA‐ScR) [23]. Ethics approval is not required for the proposed scoping review given that all resources used are publicly available.

### 3.1 Search Strategy

We have developed a search strategy in consultation with a librarian to identify published and peer‐reviewed journal articles relating to the applications of ML to HIV prognostic medicine. Articles will be considered if they are published between 1983 and 2025. This time frame has been set as HIV was identified in 1983 [24]. We will search Embase, Web of Science, Medline (PubMed), Scopus, IEEE Xplore, and ACM Digital Library from 1983 to present. Our Medline search strategy is detailed in Appendix A (Table A1). The search syntax will be adapted for use in each database. Despite the formatting adjustments, the terms and Boolean operators will remain consistent to ensure the search strategy is reproducible.

We will include peer‐reviewed articles published between 1983 and 2025 in English that discuss ML in relation to HIV prognosis. We will exclude articles (1) investigating the use of ML to evaluate the prognosis of any disease other than HIV, (2) articles investigating HIV prognosis via methods alternative to ML algorithms, (3) abstracts, posters, book reviews, blog posts, and gray literature.

### 3.2 Study Selection

After the initial search has been conducted, all studies will be uploaded to Rayyan: AI‐Powered Systematic Review Management Platform and any duplicates will be removed. The initial screening will evaluate the relevance of article titles and abstracts. Articles will be retained if they meet eligibility requirements. Articles for which the relevance is not immediately clear from screening the title or abstract will move on to full‐text screening. Full‐text screening will be carried out using Covidence: Literature Review Software by two reviewers working independently. Both Rayyan and Covidence were used to enhance the efficiency of the study selection process. The interface of Rayyan allows for rapid title and abstract screening, whereas Covidence is well‐suited for collaboration between reviewers.

We will pilot test our screening forms with a sample of 20 abstracts to ensure consistency within the screening of both reviewers. Interrater reliability will be evaluated using the *κ* statistic [25]. A minimum consensus of 80% (or a *κ* statistic of 0.8) between reviewers will be required to proceed. After the initial title and abstract screening, retained articles will undergo full‐text screening. Following the full‐text screening, reviewers will complete data extraction on the remaining retained articles. If there are disagreements between reviewers pertaining to whether an article is eligible to be included within the study, they will be resolved through discussion or by involving additional reviewers. If any systematic or scoping reviews are identified during the screening process, references from the review should be screened and any relevant studies will be added to the review if not already captured by the initial search. The study selection process will be reported and presented in a PRISMA‐ScR flow diagram.

### 3.3 Data Extraction

We will pilot test our data extraction form with a sample of 10 articles to ensure consistency between reviewers. Two independent reviewers will extract data in duplicate using the pilot tested and refined form. We will extract bibliometric details (authors, country, institutions, funding, year of publication), study objectives, study design, population, data collection, type of ML methodology, model target, outcome measures, and key findings. A draft of the data extraction form is detailed in Appendix A (Table A2). If there are disagreements between reviewers regarding the data extraction form, they will be resolved through discussion or by involving additional reviewers.

### 3.4 Analysis and Reporting

All articles included within the review will be presented in a table which will summarize author names, year of publication, location of research, aim of study, methodology, and results. The data extracted from the literature will be analyzed quantitatively to facilitate investigation into statistics regarding geographic location, year of publication, aim of research, and findings. These results will be presented in tables, charts or figures in addition to the narrative summary in order to more comprehensively report the findings of the analyses. Based on these findings, a narrative summary will further investigate trends, similarities and differences in the aforementioned components of the included studies. The narrative summary will be conducted through thematic analysis. Extracted information will be grouped into categories, which will reflect patterns and themes across the literature.

A narrative summary will follow the charted results in order to connect the data to the overarching research question on the review. Therefore, the review will aim to identify common themes among the current interventions for utilizing ML in HIV prognosis. The data will additionally inform gaps in the research and directions for future studies which will be highlighted in the review through a narrative analysis.

## 4 Discussion

The results from this scoping review will synthesize the current body of research regarding the applications of ML to HIV prognosis. In doing so, the review will highlight the ways in which ML may optimize prognostic medicine in the context of HIV care. ML has the potential to fill gaps within overworked healthcare systems, mitigating historically long wait times and rushed care. ML models show promise in bridging these gaps by providing fast, efficient, and accurate healthcare decisions.

This scoping review will identify areas where ML can enhance the efficiency of healthcare interventions by highlighting existing, relevant, and successful ML applications. Current research on the applications of ML to nuanced aspects of HIV prognosis and coinfection could inform the development of a larger‐scale model. Furthermore, the review will highlight gaps in ML research and suggest areas for future studies. As a result, the review aims to initiate the development of a widely used ML model for determining the prognosis of a person living with HIV. Namely, the review will lay the foundation for future research and ML product development in the context of HIV care.

ML is an increasingly prevalent area of healthcare research, with the number of related studies published in academic journals growing by an average of 26.97% per year as of January 2025 [26]. Additionally, given the growing global population, healthcare systems are experiencing increasing strain and overload. The combined effects of these factors are rapidly advancing healthcare systems into the era of artificial intelligence, making preliminary research essential for guiding future interventions. This review will contribute to the growing body of research in offering insight into the novel applications of ML to HIV prognostics.

### 4.1 Limitations

A limitation of this review is that it does not assess the quality of the data sets used to train the ML models identified in the literature search. In order for a ML model to be universally applicable the data set used in the training process must be large, high‐quality, and diverse [27]. To mitigate this limitation, the data extraction process will highlight the population on which each study was conducted.

Given the nature of the studies we are including, there is some risk of publication bias, but we are unable to anticipate its direction or magnitude. Within the field of ML research, the literature disproportionately represents models with strong predictive performance, while studies reporting models with null results are underrepresented. This issue is further compounded by heterogeneity in model development and reporting, which makes it difficult to compare results across studies. Therefore, there is the potential for an overestimation of ML model performance within the field of prognostic modeling. This limitation will be acknowledged in the full scoping review, and to mitigate its impact, model performance metrics will be categorized and described rather than combined, ensuring that variability across studies is accurately represented.

### 4.2 Ethical Considerations and Dissemination

Ethics approval is not required for this review as the study does not require the involvement of any participants. Only secondary data will be included in the review, all of which will be sourced from publicly available journals. Dissemination will be done through conference presentation, publication in a peer‐reviewed open‐access journal, and dissemination among researchers and physicians to promote the adoption of ML models to determine HIV prognosis.

## Author Contributions

**Sadie Gilliland:** conceptualization, investigation, writing – original draft, methodology, writing – review and editing. **Lawrence Mbuagbaw:** methodology, supervision, writing – review and editing. All authors have read and approved the final version of the manuscript. Lawrence Mbuagbaw had full access to all of the data in this study and takes complete responsibility for the integrity of the data and the accuracy of the data analysis.

## Disclosure

Sadie Gilliland affirms that this manuscript is an honest, accurate, and transparent account of the study being reported; that no important aspects of the study have been omitted; and that any discrepancies from the study as planned (and, if relevant, registered) have been explained.

## Ethics Statement

This study does not involve human participants or animals and therefore did not require ethics approval.

## Conflicts of Interest

The authors declare no conflicts of interest.

## Data Availability

This is a study protocol and therefore no data have been collected. Upon completion of the proposed scoping review, data will be made available.

## 1

**Table A1:** Search strategy

#	Query
1	artificial intelligence/machine learning/or deep learning/or supervised machine learning/or support vector machine/
2	(“artificial intelligence” or “deep learning” or “supervised machine learning” or “transfer learning” or “support vector machine”).tw,kf.
3	prognosis/or nomograms/or treatment outcome/
4	(“prognos*” or “pfactor*” or “nomograms” or “disease progression” or “treatment outcome*” or “mortality” or “survival” or “disease progression” or “clinical progression” or “clinical course” or “treatment outcome” or “clinical efficacy” or “patient‐relevant outcome*” or “hospitalization” or “length of stay” or “adherence”).tw,kf.
5	hiv/or hiv‐1/or hiv‐2/
6	(“HIV” or “HIV‐1” or “HIV‐2” or “acquired immunodeficiency syndrome” or “human T cell lymphotropic virus type III” or “HTLV‐III”).tw,kf.
7	1 or 2
8	3 or 4
9	5 or 6
10	7 and 8 and 9

*Note:* Ovid MEDLINE(R) ALL.

**Table A2:** Proposed data extraction framework.

	Category	Description
Bibliometric information	Authors	Names of all authors
	Country	Country of primary affiliation for all authors
	Institution	Institutions in which research took place
	Funders	Indication where funding for the study was sourced from
	Year of publication	The year the research was published
Purpose	Study objectives	Stated aim of the study
Methodology	Study design	Indication of whether study was of the quantitative, qualitative, or mixed methods subcategory. Indication of the specific study design within the subcategory (e.g., case control, randomized controlled trial, etc.).
	Population	Eligibility criteria for participation in the study
	Population size	Number of participants involved in the study
	Data collection and evaluation tools	Tools developed or adapted specifically for this study
	Machine learning methodology	Types of machine learning methods used
	Model target	The target of the deep learning model (i.e., classification or prediction)
	Outcome measures	Indication of how the objectives of the study were measured. Include for both primary and secondary outcomes where applicable.
Results	Reported outcomes	Key outcomes relevant to the outcomes of the scoping review
	Key findings	Summary of the key findings relevant to the applications of ML to HIV prognosis

## References

[1] World Health Organization , “HIV and AIDS,” World Health Organization.

[2] L. G. Bekker , C. Beyrer , N. Mgodi , et al., “HIV Infection,” Nature Reviews Disease Primers 9, no. 1 (2023): 42. 10.1038/s41572-023-00452-3 37591865

[3] S. E. Kellerman , J. S. Lehman , A. Lansky , et al., “HIV Testing Within At‐Risk Populations in the United States and the Reasons for Seeking or Avoiding HIV Testing,” JAIDS Journal of Acquired Immune Deficiency Syndromes 31, no. 2 (2002): 202–210. 12394799 10.1097/00126334-200210010-00011

[4] A. Mateo‐Urdiales , S. Johnson , R. Smith , J. B. Nachega , and I. Eshun‐Wilson , “Rapid Initiation of Antiretroviral Therapy for People Living With HIV,” Cochrane Database of Systematic Reviews 2019, no. 6 (2019): CD012962. 10.1002/14651858.CD012962.pub2 PMC6575156 31206168

[5] A. Ankomah , J. K. Ganle , M. Y. Lartey , et al., “ART Access‐Related Barriers Faced by HIV‐Positive Persons Linked to Care in Southern Ghana: A Mixed Method Study,” BMC Infectious Diseases 16, no. 1 (2016): 738. 27927183 10.1186/s12879-016-2075-0 PMC5142337

[6] F. Nakagawa , R. K. Lodwick , C. J. Smith , et al., “Projected Life Expectancy of People With HIV According to Timing of Diagnosis,” AIDS 26, no. 3 (2012): 335–343. 22089374 10.1097/QAD.0b013e32834dcec9

[7] S. Qin , J. Lai , H. Zhang , et al., “Predictive Factors of Viral Load High‐Risk Events for Virological Failure in HIV/AIDS Patients Receiving Long‐Term Antiviral Therapy,” BMC Infectious Diseases 21, no. 1 (2021): 448. 34006230 10.1186/s12879-021-06162-z PMC8130293

[8] L. Mbuagbaw , S. Fernando , C. Lee , M. Owino , C. Youssef , and M. E. Snow , “Barriers and Facilitators to Improving the Cascade of HIV Care in Ontario: A Mixed Method Study,” BMC Health Services Research 24, no. 1 (2024): 48. 38200516 10.1186/s12913-023-10481-z PMC10782539

[9] N. Riccardi , G. A. Rotulo , and E. Castagnola , “Definition of Opportunistic Infections in Immunocompromised Children on the Basis of Etiologies and Clinical Features: A Summary for Practical Purposes,” Current Pediatric Reviews 15, no. 4 (2019): 197–206. 31242834 10.2174/1573396315666190617151745 PMC7040525

[10] H. M. Naif , “Pathogenesis of HIV Infection,” Infectious Disease Reports 5, no. 11 (2013): e6. 10.4081/idr.2013.s1.e6 PMC3892619 24470970

[11] C. Zhang , X. Li , Y. Liu , et al., “Co‐Infections of Tuberculosis, Hepatitis B or C Viruses in a Cohort of People Living With HIV/AIDS in China: Predictors and Sequelae,” AIDS Care 29, no. 8 (2017): 974–977. 27998171 10.1080/09540121.2016.1271388 PMC8170663

[12] Q. Bi , K. E. Goodman , J. Kaminsky , and J. Lessler , “What Is Machine Learning? A Primer for the Epidemiologist,” American Journal of Epidemiology 188, no. 12 (2019): 2222–2239. 31509183 10.1093/aje/kwz189

[13] H. Habehh and S. Gohel , “Machine Learning in Healthcare,” Current Genomics 22, no. 4 (2021): 291–300. 35273459 10.2174/1389202922666210705124359 PMC8822225

[14] S. Chen , J. Yu , S. Chamouni , Y. Wang , and Y. Li , “Integrating Machine Learning and Artificial Intelligence in Life‐Course Epidemiology: Pathways to Innovative Public Health Solutions,” BMC Medicine 22, no. 1 (2024): 354. 39218895 10.1186/s12916-024-03566-x PMC11367811

[15] D. Bzdok , M. Krzywinski , and N. Altman , “Machine Learning: A Primer,” Nature Methods 14, no. 12 (2017): 1119–1120. 29664466 10.1038/nmeth.4526 PMC5905345

[16] S. Ustebay , A. Sarmis , G. K. Kaya , and M. Sujan , “A Comparison of Machine Learning Algorithms in Predicting COVID‐19 Prognostics,” Internal and Emergency Medicine 18, no. 1 (2023): 229–239. 36116079 10.1007/s11739-022-03101-x PMC9483274

[17] J. Fieggen , E. Smith , L. Arora , and B. Segal , “The Role of Machine Learning in HIV Risk Prediction,” Frontiers in Reproductive Health 4 (2022): 1062387. 36619681 10.3389/frph.2022.1062387 PMC9815547

[18] J. L. Marcus , W. C. Sewell , L. B. Balzer , and D. S. Krakower , “Artificial Intelligence and Machine Learning for HIV Prevention: Emerging Approaches to Ending the Epidemic,” Current HIV/AIDS Reports 17, no. 3 (2020): 171–179. 32347446 10.1007/s11904-020-00490-6 PMC7260108

[19] J. L. Marcus , L. B. Hurley , D. S. Krakower , S. Alexeeff , M. J. Silverberg , and J. E. Volk , “Use of Electronic Health Record Data and Machine Learning to Identify Candidates for HIV Pre‐Exposure Prophylaxis: A Modelling Study,” Lancet HIV 6, no. 10 (2019): e688–e695. 31285183 10.1016/S2352-3018(19)30137-7 PMC7152802

[20] B. Zhan , W. Wei , Z. Xie , et al., “Machine Learning‐Based Prognostic Prediction for Hospitalized HIV/AIDS Patients With *Cryptococcus* Infection in Guangxi, China,” BMC Infectious Diseases 24, no. 1 (2024): 1121. 39379851 10.1186/s12879-024-10013-y PMC11459777

[21] A. Rivero‐Juárez , D. Guijo‐Rubio , F. Tellez , et al., “Using Machine Learning Methods to Determine a Typology of Patients With HIV‐HCV Infection to be Treated With Antivirals,” *PLoS One* 15, no. 1 (2020): e0227188. 10.1371/journal.pone.0227188 PMC6953863 31923277

[22] M. C. Prosperi , S. Di Giambenedetto , I. Fanti , et al., “A Prognostic Model for Estimating the Time to Virologic Failure in HIV‐1 Infected Patients Undergoing a New Combination Antiretroviral Therapy Regimen,” BMC Medical Informatics and Decision Making 11, no. 1 (2011): 40. 21672248 10.1186/1472-6947-11-40 PMC3144446

[23] A. C. Tricco , E. Lillie , W. Zarin , et al., “PRISMA Extension for Scoping Reviews (PRISMA‐ScR): Checklist and Explanation,” Annals of Internal Medicine 169, no. 7 (2018): 467–473. 30178033 10.7326/M18-0850

[24] L. Montagnier , “A History of HIV Discovery,” Science 298, no. 5599 (2002): 1727–1728. 12459575 10.1126/science.1079027

[25] M. L. McHugh , “Interrater Reliability: The Kappa Statistic,” Biochemia Medica 22, no. 3 (2012): 276–282. 23092060 PMC3900052

[26] Y. Xie , Y. Zhai , and G. Lu , “Evolution of Artificial Intelligence in Healthcare: A 30‐Year Bibliometric Study,” Frontiers in Medicine 11 (2025): 1505692. 39882522 10.3389/fmed.2024.1505692 PMC11775008

[27] M. I. Ahmed , B. Spooner , J. Isherwood , M. Lane , E. Orrock , and A. Dennison , “A Systematic Review of the Barriers to the Implementation of Artificial Intelligence in Healthcare,” Cureus 15, no. 10 (2023): e46454. 37927664 10.7759/cureus.46454 PMC10623210

